# Lines to a Skeleton—Poetry

**Published:** 1873-01

**Authors:** 


### LINES TO A SKELETON.

[The following poem was found near a skeleton in the Museum of the Royal College of Surgeons, Lincoln's Inn, London, and was sent for publication to the *Morning Chronicle*. Though fifty guineas reward was offered for the discoovery of the author, his name has never transpired.]

Behold this ruin! 'Twas a skull,
Once of ethereal spirit full;
This narrow cell was life's retreat,
This space was thought's mysterious seat.
What beauteous visions filled this spot!
What dreams of pleasure long forgot!
Nor hope, nor love, nor joy, nor fear,
Have left one trace or record here.

Beneath that mouldering canopy
Once shone the bright and busy eye.
But start not at the dismal void:
If social love that eye employed.
If with no lawless fire it gleamed,
But through the dew of kindness beamed,
That eye shall be forever bright
When stars and suns are sunk in night.

Within this hollow cavern hung
The ready, swift and tuneful tongue.
If falsehood's honey it disdained,
And when it could not praise, was chained,
If bold in virtue's cause it spoke,
Yet gentle concord never broke,
That silent tongue shall plead for thee
When time unveils eternity.

Say, did those fingers delve the mine!
Or with its envied rubies shine!
To hew the rock or wear the gem,
Can little now avail to them,
But if the page of truth they sought,
Or comfort to the mourner brought,
The hands a richer meed shall claim
Than all that wait on wealth or fame.

Avails it whether bare or shod
Those feet the paths of duty trod?
If from the halls of ease they fled
To seek affliction's humble shed,
If grandeur's guilty bribe they spurned
And home to virtue's cot returned,
Those feet with angel's ways shall vie,
And tread the palace of the sky.

